# Bioinspired Breathable Architecture for Water Harvesting

**DOI:** 10.1038/srep16798

**Published:** 2015-11-18

**Authors:** Rowan M. von Spreckelsen, Matthew T. Harris, James M. Wigzell, Rebekah C. Fraser, Andrea Carletto, Daniel P. K. Mosquin, Douglas Justice, Jas Pal S. Badyal

**Affiliations:** 1 Department of Chemistry, Science Laboratories, Durham University, Durham DH1 3LE, England, UK; 2 UBC Botanical Garden, University of British Columbia, Vancouver V6T 1Z4, Canada

## Abstract

*Thuja plicata* is a coniferous tree which displays remarkable water channelling properties. In this article, an easily fabricated mesh inspired by the hierarchical macro surface structure of *Thuja plicata* branchlets is described which emulates this efficient water collection behaviour. The key parameters are shown to be the pore size, pore angle, mesh rotation, tilt angle (branch droop) and layering (branch overlap). Envisaged societal applications include water harvesting and low cost breathable architecture for developing countries.

The United Nations estimates that over one in ten people across the world do not have access to clean water^1^. Hence, affordable, eco-sustainable methods for water collection are a major global challenge facing society today. *Thuja plicata*, a coniferous tree whose natural habitat is North Western America^2^, displays remarkable water channelling properties during torrential rainfall, where the ground underneath its extended branches remains dry (despite possessing an open structure allowing the passage of sunlight). *Thuja plicata* trees grow in a conical shape, with drooping and overlapping branches, Fig. 1(a). These branches are composed of drooping flat branchlets, characterised by a central main stem, from which side stems protrude alternately off either side at approximately 45° angles within the same plane, Fig. 2(a). Individual tines extend from these side stems parallel to the main stem comprising small leaves which are scale-like. Water flow bridging across the tines of the leaf is clearly visible to the naked eye during rainfall or water sprinkling, Fig. 1(b) and Supplementary Information Video 1. This functional role of moisture channelling by *Thuja plicata* branchlets could be an adaptive advantage for leaves in the mild, wet North Western America climate enabling them to quickly shed surface water, since liquid water is required for most fungal parasites to both germinate and reproduce (leaf blight)^3^. Scanning electron microscopy (SEM) analysis of the *Thuja plicata* leaf shows the presence of microscale grooves, within the otherwise smooth layer of the epicuticular wax, orientated parallel along the leaves and converging at the tip (these grooves might also assist water channelling), Fig. 1(c). No major difference in surface structure was evident on the microscale between the leaf surfaces facing the adaxial versus the abaxial sides of the branchlet (which is consistent with their similar water stream collection efficiencies, Table 1). A comparison between freshly cut and aged branchlet samples indicated that any degradation of the surface (chemistry) does not lead to a significant variation in water stream collection efficiency. Neither did altering leaf wettability by surface functionalisation (hydrophilic—hydrophobic water contact angle ranging between 35–122°, Supplementary Information Table 1).

Replication of the flat *Thuja plicata* branchlet was accomplished by 3D printing its macro structure into a uniform mesh (thereby providing a more robust and practical structure instead of just fabricating the branchlets directly), and demonstrating comparable liquid stream collection efficiencies across a range of surface wettabilities (hydrophilic—hydrophobic water contact angle ranging between 65–132°, Fig. 2 and Supplementary Information Tables 2–3). So long as the incident liquid stream hits the mesh structure, no significant lowering in collection efficiency occurs (remaining above 99%) with increasing pore size dimensions (x and y values for θ = 45°), Fig. 2(c).

The natural drooping of the *Thuja plicata* branchlets was investigated by varying the angle of the mesh with respect to the vertical (ϕ), Fig. 2(d). Tilting of the mesh by just 20° from the horizontal (ϕ = 70°) is sufficient to yield + 99.7% water collection efficiencies. For other probe liquids, a lowering of liquid surface tension was found to correlate to an increase in the value of ϕ at which the mesh fails (the liquid drips through). This was noted for polar liquids (water and propan-2-ol), and a non-polar liquid (decane). Below these critical ϕ angle values, the high collection efficiencies for water, propan-2-ol, and decane were measured to be very similar.

Orientation within-the-plane of the mesh (with respect to incident water flow) also governs liquid collection efficiency, Fig. 2(e). When the rotation angle within-the-plane of the mesh (β) equals 0° or 135°, water easily flows down the vertical struts of the mesh under gravity. However, as β approaches 70°, the water is forced to flow over the diamond orientation of the mesh leading to water dripping through the mesh.

The mesh internal pore angle, θ is another key parameter (pore shape), Fig. 2(f). It explains why the *Thuja plicata* branchlets have an angle, θ = 45° between the tines and the side stems, so as to minimise/prevent water dripping through the branchlets. This is in contrast to the worst case scenario (θ = 90°), where the 3D printed mesh contains square pores, and over 60% of the water falls through the mesh.

As explained above, the aforementioned water collection efficiencies rely on the water stream hitting the mesh surface, which limits the scope of potential applications for just a single mesh layer. More realistic rainfall across the whole single mesh layer (including pores) was emulated by utilising a water sprinkler, and in this case collection efficiency decreases with larger pore size due to water droplets falling directly through the holes, Fig. 3(b). This lowering in performance for sprinkled water could be overcome by utilising a double mesh layer, which effectively imitates the overlapping branchlets of the *Thuja plicata,* and led to a significant improvement in sprinkled water collection (less than 1% drip through for pore size of x = y = 3 mm). Further enhancement in water collection efficiency could be attained by reducing the separation distance between the two mesh layers, Fig. 3(c). For envisaged solar harvesting applications, a visible light transparency through the double layer of 40% correlates to less than 10% of the water sprinkled (simulated rain) dripping through (for a pore size of x = y = 7 mm), Fig. 3(d). Variants of this *Thuja plicata* inspired approach for maximising light transparency would be to 3D print the hierarchical branchlet—side stem—tine structures and assemble them onto drooping central pillars (branches), or incorporate puckering of the mesh on the macroscale so as to further mimic the channelling behaviour of *Thuja plicata* branchlet side stems.

Furthermore, it is envisaged that the pore shape of this *Thuja plicata* inspired mesh could be easily incorporated into the weave of textiles to provide waterproof, breathable fabrics for tents. Other potential applications of these bioinspired meshes include: fog harvesting nets, saline-free water collection on sailing boats, filtration, and breathable architecture (agriculture, transport, photovoltaics, residential buildings and high rise offices). For the latter, by offsetting two (or more) layers of mesh with respect to each other, the structure effectively becomes waterproof, breathable and light transparent. Such eco-friendly breathable slanted windows or roofing could help to dramatically reduce the world’s energy consumption (for instance, in hot climates where air conditioning tends to be a major drain on grid electricity and cause of power outages^4^). In the case of bioinspired roofing, a gap being manually or thermostatically opened between two mesh layers during the night when the outside temperature drops allows cool air to circulate throughout the building, whilst offering protection against rainfall. Then during the heat of the day, the gap closes to create an impermeable layer in order to retain the cool air inside. The sheer simplicity of design and fabrication of such bioinspired roofing makes it readily affordable for widespread adoption in developing countries.

## Methods

### Materials

*Thuja plicata* branchlet samples were collected from Durham University Botanical Garden.

### Scanning Electron Microscopy (SEM)

Individual branchlet specimens were prepared for scanning electron microscopy analysis using a glycerol substitution process in order to avoid structural collapse due to dehydration^5^. Each sample was then mounted onto a carbon disk supported on an aluminium stud. For high resolution images, gold coating was required in order to avoid excess surface charging. Images were taken on a scanning electron microscope (Cambridge Stereoscan 240) operating in secondary electron detection mode, in conjunction with 8 kV accelerating voltage, and a working distance between 8–26 mm.

### Surface Functionalisation

Plasmachemical surface functionalisation was carried out in a cylindrical glass chamber (5 cm diameter, 470 cm^3^ volume) enclosed within a Faraday cage^6^. This was connected to a two-stage rotary pump via a liquid nitrogen cold trap. An inductor-capacitor (L-C) impedance matching network was used to minimise the standing wave ratio (SWR) for the power transmitted from a 13.56 MHz radio frequency generator (RF) to a copper coil (10 turns, spanning 8 cm) externally wound around the glass chamber. In the case of pulsed plasma functionalisation, a signal generator was used to trigger the RF power supply, and the pulse shape monitored with an oscilloscope. Prior to each plasma treatment, the reactor was scrubbed with detergent, rinsed in propan-2-ol (99.5%, Fischer Scientific Ltd.), and further cleaned using a 50 W air plasma at 0.2 mbar pressure for 30 min. Each sample was then placed into the centre of the reactor, followed by evacuation to system base pressure (in the case of *Thuja plicata* branchlets, the system was not evacuated below 0.1 mbar in order to avoid drying out the branchlet sample). Next, precursor gas/vapour was admitted into the chamber via a needle control valve at 0.2 mbar pressure, and the electrical discharge ignited. Upon completion of plasma exposure, the gas/vapour source was turned off, and the chamber vented to atmosphere. A range of different types of plasmachemical surface functionalisation were performed in order to explore a range of wettabilities using the following precursors: air, tetramethylsilane (+99.9%, Alfa Aesar), 1H,1H,2H,2H–perfluorooctyl acrylate (+95%, Fluorochem Ltd.), and carbon tetrafluoride (+99.7%, Air Products Inc.), see Supplementary Information Table 2.

### Static Water Contact Angle Measurement

Microlitre sessile droplet contact angle analysis was undertaken at 20 °C with a video contact angle goniometer (VCA 2500 XE, AST Products Ltd) using a 1.0 μL droplet of high purity water (BS 3978 grade 1). Static contact angle measurement was taken after 3 s and there was no visible change in the droplet shape during this period.

### Water Stream Collection

**Figure 2:**
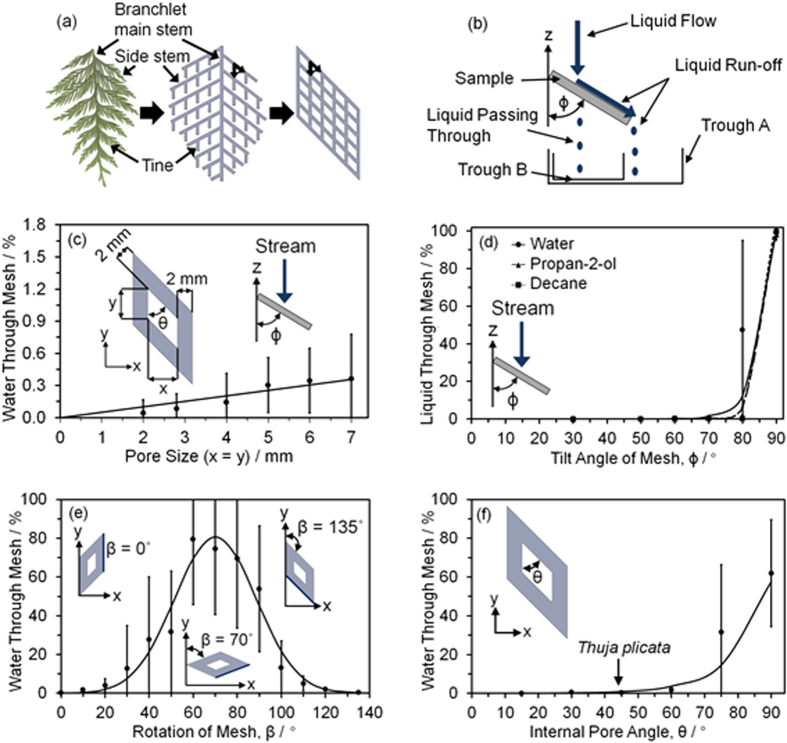
3D printed replication. (**a**) Bioinspired water channelling mesh structure based upon the *Thuja plicata* branchlet. (**b**) Apparatus for measurement of water stream collection efficiency. (**c**) Water stream collection efficiency of 3D printed meshes as a function of pore size dimensions x and y (x = y, θ = 45°, ϕ = 50°, and β = 0°) (see Supplementary Information Video 2). (**d**) Liquid stream collection efficiency of the 3D printed mesh as a function of mesh tilt angle, ϕ (x = y = 4 mm, θ = 45°, and β = 0°) for water, propan-2-ol, and decane (surface tension = 72.8 mN m^−1^, 21.3 mN m^−1^, and 23.8 mN m^−1^ respectively), where the much larger error value at ϕ = 80° is due to the transition region between the water running completely off the mesh and starting to drip through the mesh. (**e**) Water stream collection efficiency of a tilted 3D printed mesh as a function of rotation within x-y plane of the tilted mesh with respect to incident water flow, β (x = y = 4 mm, θ = 45°, and ϕ = 60°). (**f**) Water stream collection efficiency of 3D printed meshes as a function of the internal pore angle, θ (x = y = 4 mm, ϕ = 70°, and β = 0°). Error bars in all cases are ± 1 standard deviations and, if absent, are smaller than shown symbol size.

Tap water, propan-2-ol (Fischer Scientific Ltd.), and decane (Aldrich Inc.) were employed as test liquids. A filled 25.0 ml burette (average water flow rate = 814 ± 5 μl s^−1^, diameter of burette outlet = 0.5 mm) was used to provide a liquid stream incident upon the sample mounted at an angle, ϕ, to the vertical, Fig. 2(b). The stream was directed to hit the sample surface with an impact cross-section of 1.96 mm^2^. Any liquid dripping through the sample was measured by volume of liquid collected in trough B (4.7 cm diameter 100 ml beaker), Fig. 2(b). The volume of water channelled into trough A (14 cm diameter crystallisation dish), combined with that measured for trough B, enabled the liquid residue remaining on the sample and emptied collection troughs to be calculated. These measurements were then used to determine the water stream collection efficiency by calculating the percentage of liquid which had dripped through the sample. Each data point was repeated at least 10 times.

### Sprinkled Water Collection

**Figure 3:**
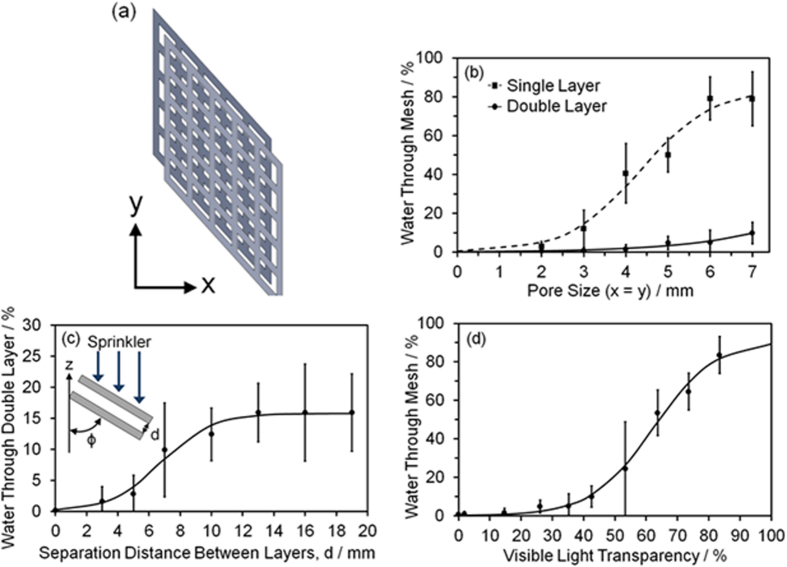
Mesh layering. (**a**) Schematic showing the offset configuration of the double layer (where the junctions of one layer are positioned over the centre of the pores of the other layer). (**b**) Water sprinkler collection efficiencies of single and double layer 3D printed meshes as a function of the mesh pore size (x = y, θ = 45°, ϕ = 70°, β = 0°, and d = 3 mm) (see Supplementary Information Video 3). (**c**) Water sprinkler collection efficiency of the double layer of mesh as a function of the separation distance, d, between the mesh layers (x = y = 4 mm, θ = 45°, ϕ = 70°, and β = 0°). (**d**) Water sprinkler collection efficiency of the double mesh layer as a function of the percentage of visible light transparency through the mesh layers with increasing pore size (x = y, θ = 45°, ϕ = 70°, β = 0°, and d = 3 mm). The large error shown in the amount of water passing through the mesh at 53% light transparency is due to the transition region between the water running completely off the mesh and passing through the mesh. Error bars in all cases are ± 1 standard deviation and, if absent, are smaller than shown symbol size.

Rain impact onto the sample was emulated using a 60 ml syringe attached to a sprinkler head (water flow rate = 3.7 ± 0.3 ml s^−1^). The sprinkler head contained 7 holes (1 mm diameter) equally spaced over an area of 9.62 cm^2^. The volume of water channelled into trough A (26 cm × 26 cm × 6 cm crystallisation dish), combined with that measured for trough B (6.9 cm diameter 250 ml beaker), enabled the liquid residue remaining on the sample and emptied collection troughs to be calculated, Fig. 2(b). These measurements were then used to determine the sprinkled water collection efficiency by calculating the percentage of liquid which had dripped through the sample. Each data point was repeated at least 10 times. For the double mesh layer measurements, the layers were offset so that junctions of one mesh were centred over the pores of the other mesh, Fig. 3(a).

### 3D Printing

The meshes were designed using Solidworks computer software (Dassault Systèmes SolidWorks Corp.) and 3D printed from an acrylic compound (Objet Fullcure 720 (RGD720), Statasys Ltd.) using a 3D printer (Objet Eden 500V, Stratasys Ltd.)

## Additional Information

**How to cite this article**: von Spreckelsen, R. M. *et al.* Bioinspired Breathable Architecture for Water Harvesting. *Sci. Rep.* **5**, 16798; doi: 10.1038/srep16798 (2015).

## Supplementary Material

Supplementary Information

Supplementary Video 1

Supplementary Video 2

Supplementary Video 3

## Figures and Tables

**Figure 1:**
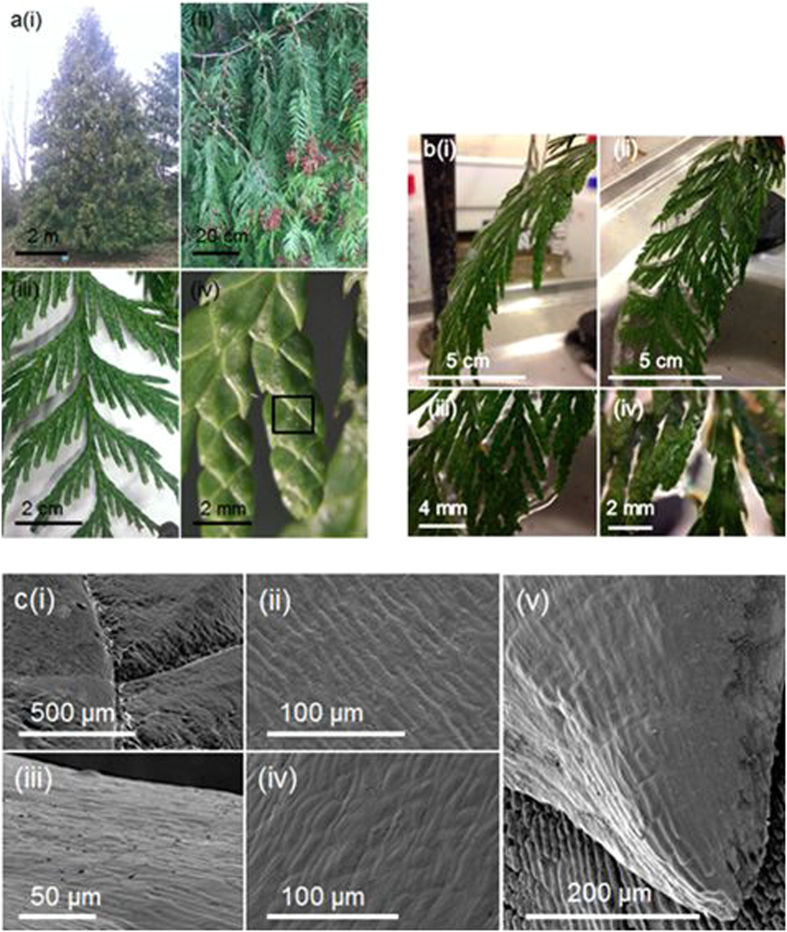
Water channelling by *Thuja plicata*. (**a**) Optical images of *Thuja plicata*: (i) the tree; (ii) overlapping, drooping branches; (iii) a single flat branchlet; and (iv) individual tines consisting of flat, overlapping scale-like leaves (where the outlined square corresponds to the SEM scan area shown in Fig. 1(c) (i) of overlapping leaves). (**b**) Optical images showing incident water stream bridging across the adaxial *Thuja plicata* tines (see Supplementary Information Video 1): (i) side-on view showing the water flowing over the branchlet surface; (ii) front-on view showing the water flowing over the branchlet surface; and (iii-iv) zoomed-in views showing water bridging across the tines. (**c**) Scanning Electron Microscopy (SEM) micrographs of *Thuja plicata* branchlet leaves: (i–iii) adaxial surface at increasing magnification, where (i) corresponds to the outlined square inset in Fig. 1(a)(iv) for overlapping leaves; (iv) abaxial surface; and (v) adaxial surface leaf tip. All images were taken by the authors.

**Table 1:** Water channelling by *Thuja plicata.*

Sample	Leaf Static Water Contact Angle/°		Water through Branchlet Surface/%	
	Adaxial	Abaxial	Adaxial	Abaxial
Fresh	69 ± 9	86 ± 9	0.3 ± 0.6	0.2 ± 0.3
Aged	81 ± 6	86 ± 8	0.3 ± 0.4	0.9 ± 1.0

Comparison of water contact angle and water stream collection efficiency of a freshly cut *Thuja plicata* branchlet and an aged *Thuja plicata* branchlet (cut and the main stem immersed in water for five days, then removed from water and air dried for a further three days prior to analysis). Error values in all cases are ±1 standard deviation.
