# Supplementary material for: Bioinspired Breathable Architecture for Water Harvesting
Source: Sci Rep. 2015 Nov 18;5:16798. doi: 10.1038/srep16798 (PMC4649541; doi:10.1038/srep16798)
Supplement: Supplementary Information [file srep16798-s1.pdf]

Bioinspired Breathable Architecture for Water Harvesting

(SUPPLEMENTARY INFORMATION)

Rowan M. von Spreckelsen[†], Matthew T. Harris[†], James M. Wigzell[†], Rebekah C. Fraser[†], Andrea Carletto[†], Daniel P. K. Mosquin[§], Douglas Justice[§], and Jas Pal S. Badyal[†*]

† Department of Chemistry, Science Laboratories, Durham University, Durham DH1 3LE, England, UK

§ UBC Botanical Garden, University of British Columbia, Vancouver V6T 1Z4, Canada.

* Corresponding author email: j.p.badyal@durham.ac.uk

Supplementary Information Video 1: Front-on view of water stream flowing over *Thuja plicata* branchlet surface.

Supplementary Information Video 2: Side-on view of water stream flowing over a 3D printed single layer mesh ($x = y = 3$ mm, $\theta = 45^\circ$, $\phi = 60^\circ$, and $\beta = 0^\circ$).

Supplementary Information Video 3: Side-on view of sprinkled water flowing over double layer of 3D printed meshes ($x = y = 3$ mm, $\theta = 45^\circ$, $\phi = 60^\circ$, $\beta = 0^\circ$, and $d = 3$ mm, where the junctions of one mesh layer was centred over the pores of the other mesh layer).

Supplementary Information Table 1: Role of surface chemistry on *Thuja plicata* branchlet surfaces: Static water contact angle and water stream collection efficiencies of adaxial *Thuja plicata* branchlet surfaces before and after plasmachemical surface functionalization. The averaged static water contact angle of *Thuja plicata* leaves is quoted in all cases before surface functionalization (the same throughout). Whilst, the water stream collection efficiency values are reported for the individual respective functionalised leaves. The plasmachemical surface functionalization precursors employed were: air, tetramethylsilane (TMS)[1], and 1H,1H,2H,2H–perfluorooctyl acrylate (PFAC–6)[2] as described in Methods. The error values quoted are 1 standard deviation.

Plasmachemical Surface Functionalisation	Leaf Static Water Contact Angle / °		Water Through Adaxial Surface of Branchlet / %	
	Before	After	Before	After
Air	69 ± 9	35 ± 9	0.4 ± 0.8	0.4 ± 0.5
TMS	69 ± 9	105 ± 5	0.7 ± 1.0	0.5 ± 0.7
PFAC–6	69 ± 9	122 ± 6	0.4 ± 0.5	0.5 ± 0.8

Supplementary Information Table 2: Plasmachemical surface functionalisation: Experimental parameters employed for plasmachemical surface functionalisation using the following precursors: air, tetramethylsilane (TMS), 1H,1H,2H,2H–perfluorooctyl acrylate (PFAC–6), and CF_4.

Precursor	Power / W	Pressure / mbar	Duration / min
Air	20 (CW)	0.2	1
TMS	3 (CW)	0.2	3
PFAC–6	50 (Pulsed†)	0.2	10
CF_4	5 (CW)	0.2	1

†50 W peak power, t_{on} = 20 µs, and t_{off} = 20 ms.

Supplementary Information Table 3: Role of surface chemistry for 3-D printed meshes: Static water contact angle and water stream collection efficiencies of plasmachemical surface functionalized 3-D printed meshes ($x = y = 4$ mm, $\theta = 45^\circ$, $\phi = 60^\circ$, and $\beta = 0^\circ$). The plasmachemical surface functionalization precursors employed were air for hydrophilic and CF_4 gas for hydrophobic[3]. The errors quoted are 1 standard deviation.

**Plasma Surface Functionalisation**	**3-D Printed Mesh Water Contact Angle / °**	**Water Through 3-D Printed Mesh / %**
Non-Functionalised	93 ± 8	0 ± 0
Air	65 ± 5	0 ± 0
CF_4	132 ± 3	0.3 ± 0.4†

† This is attributable to a small amount of water droplet bouncing off the hydrophobic surface during the initial water droplet impact (prior to the onset of continuous water stream formation).

Supplementary Information References

1 Fonseca, J.L.C., Apperley, D.C. & Badyal, J. P. S. Plasma Polymerization of Tetramethylsilane. *Chem. Mater.* **5**, 1676-1682 (1993).

2 Coulson, S.R., Woodward, I.S., Brewer, S.A., Willis, C. & Badyal, J. P. S. Ultra-Low Surface Energy Plasma Polymer Films. *Chem. Mater.* **12**, 2031-2038 (2000).

3 Hopkins, J. & Badyal, J. P. S. Non-equilibrium Glow Discharge Fluorination of Polymer Surfaces. *J. Phys. Chem.* **99**, 4261-4264 (1995).
